# How to Treat Hepatocellular Carcinoma in Elderly Patients

**DOI:** 10.3390/ph14030233

**Published:** 2021-03-08

**Authors:** Piera Federico, Emilio Francesco Giunta, Annalisa Pappalardo, Andrea Tufo, Gianpaolo Marte, Laura Attademo, Antonietta Fabbrocini, Angelica Petrillo, Bruno Daniele

**Affiliations:** 1 Medical Oncology Unit, Ospedale del Mare, 80147 Napoli, Italy; emiliofrancescogiunta@gmail.com (E.F.G.); annalisa.pappalardo88@gmail.com (A.P.); laura.attademo@gmail.com (L.A.); antonietta.fabbrocini@gmail.com (A.F.); angelic.petrillo@gmail.com (A.P.); b.daniele@libero.it (B.D.); 2 Department of Precision Medicine, School of Medicine, University of Study of Campania “L. Vanvitelli”, 80131 Napoli, Italy; 3 Surgical Unit, Ospedale del Mare, 80147 Napoli, Italy; tufo.andrea@gmail.com (A.T.); gianpaolo.marte@gmail.com (G.M.)

**Keywords:** geriatric assessment, geriatric scale, target therapy, first-line, second-line, transplant, resection, systemic treatment

## Abstract

Hepatocellular carcinoma (HCC) is the primary tumour of the liver with the greatest incidence, particularly in the elderly. Additionally, improvements in the treatments for chronic liver diseases have increased the number of elderly patients who might be affected by HCC. Little evidence exists regarding HCC in old patients, and the elderly are still underrepresented and undertreated in clinical trials. In fact, this population represents a complex subgroup of patients who are hard to manage, especially due to the presence of multiple comorbidities. Therefore, the choice of treatment is mainly decided by the physician in the clinical practice, who often tend not to treat elderly patients in order to avoid the possibility of adverse events, which may alter their unstable equilibrium. In this context, the clarification of the optimal treatment strategy for elderly patients affected by HCC has become an urgent necessity. The aim of this review is to provide an overview of the available data regarding the treatment of HCC in elderly patients, starting from the definition of “elderly” and the geriatric assessment and scales. We explain the possible treatment choices according to the Barcelona Clinic Liver Cancer (BCLC) scale and their feasibility in the elderly population.

## 1. Introduction

Hepatocellular carcinoma (HCC) is the primary tumour of the liver with the greatest incidence worldwide, and almost 13,000 new diagnoses of HCC are expected in Italy in 2020 [1,2]. It arises especially in patients with previous liver diseases (cirrhosis from viral infections, alcohol abuse, etc.), and the risk of developing HCC increases with age, reaching the highest incidence in the seventh decade of life. Despite therapeutic advances, the prognosis of HCC is severe, especially in patients with advanced disease [1,3]. For this reason, each HCC patient requires a careful and global evaluation in dedicated and high-volume centers by a multidisciplinary team. This team should include oncologists, radiologists, radiotherapists, surgeons, nutritionists and gastroenterologists due to the complexity of the management of HCC patients, who often show liver impairment, gastrointestinal bleeding, ascites or hepato-renal syndrome due to cirrhosis, and/or cachexia and malnutrition also due to the alcohol abuse. Each patient should be carefully staged, and even if multiple staging systems do exist, the Barcelona Clinic Liver Cancer (BCLC) classification is the most used in Europe [4,5].

The majority of patients affected by HCC are elderly, and age is one of the risk factors in HCC development. Improvements in the treatments of chronic liver diseases in recent decades have increased life expectations and the number of elderly patients who develop HCC. Therefore, a comprehensive geriatric assessment could be useful for oncologists to distinguish between fit and frail patients and to choose patients eligible to receive an active treatment. However, elderly patients are underrepresented in clinical trials [6], and this has led to undertreatment of these patients in clinical practice. In fact, they also represent a hard-to-manage, complex subgroup of patients due to the presence of multiple comorbidities. Therefore, since there is a lack of recommendations regarding HCC in the elderly, the choice of the treatment is mainly decided by each physician in the clinical practice, who often does not compare them to identify an active treatment that best avoids the possibility of drug-related adverse events (AEs), which may alter their unstable balance.

Based on this background, the clarification of the optimal treatment strategy for elderly patients with HCC has become a pressing need. The aim of this narrative review is to provide an overview of the available data regarding the treatment of HCC in elderly patients, starting from the definition of “elderly” and the geriatric assessment and scales. Then, we explain the possible treatment choices according to the BCLC algorithm and their feasibility in the elderly population.

## 2. Geriatric Assessment and Scales: A Tool to Choose a Tailored Treatment for Elderly HCC Patients

The definition of “elderly patient” is a controversial issue [7], due to the fact that chronological age alone cannot describe the complexity of biological events that drive the ageing process. Chronological age is a simple way of defining a target population and, according to the International Society of Geriatric Oncology (SIOG), 70 years is the cut-off currently used to define a patient as elderly [8]. Accordingly, the scientific literature on liver disease, including HCC, refers to 70 years old as the cut-off to distinguish elderly and non-elderly patients [9].

More than 60% of patients who are newly diagnosed with cancer are ≥70 years of age; this finding, along with the trend of ageing of the global population, requires a better definition of the correct approach to these patients [10]. Therefore, the challenge for oncologists is to estimate the risk-benefit ratio, meaning to define when the expected benefit of treatment is superior to the risk of toxicity and/or dose reduction/discontinuation of treatment in the elderly population, which has a limited life expectancy and a reduced ability to adapt to physical and psychological stress. This evaluation could be difficult, and the routine clinical history and the standard performance status (PS) scales, which are used daily in clinical practice (such as Karnofsky Performance Status (KPS) or Eastern Cooperative Oncology Group Performance Status (ECOG PS)), are often not adequate to evaluate the “individual reserve” and to differentiate the frail patient from the fit patient.

In this context, several tools, such as the Chemotherapy Risk Assessment Scale for High-Age Patients (CRASH) and the Cancer and Aging Research Group (CARG) chemotherapy toxicity calculator, have been developed to assist oncologists in the prediction of chemotherapy tolerance in this population [11]. According to American Society of Clinical Oncology (ASCO) guidelines, a comprehensive geriatric assessment (CGA) should be performed in all the older patients diagnosed with cancer before starting treatment [4,8,12].

**Figure 1:**
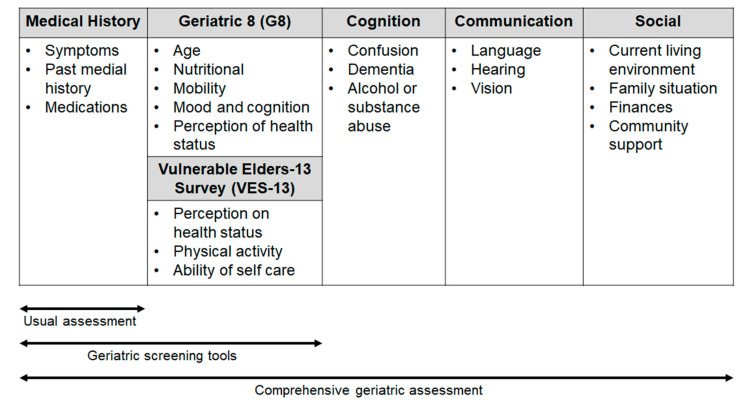
Areas of investigation used in the geriatric scales and assessment.

Comprehensive geriatric assessment (CGA) includes an evaluation of several issues (comorbidity, polypharmacy, functional status and nutritional status, psychological health, family and social support, cognition; see Figure 1) with the use of different tests (e.g., Activities of Daily Living, Mini Mental State Examination, Geriatric Depression Scale, Mini Nutritional Assessment), that can help to predict the adverse events (AEs) of treatment and the patient outcome.

To meet the needs of selected patients in the daily clinical practice, in 2005, SIOG recommended for the first time the use of a brief and simple screening test in the elderly, with the aim of identifying those patients with “a geriatric risk profile” for which CGA is necessary [8]. Therefore, in recent years, a few screening tools have been developed and validated [8]. Some consist of selected questions from validated geriatric scales (e.g., the Geriatric Depression Screen and Mini-Mental Status Exam, abbreviated CGA), whereas others, such as the Geriatric 8 (G8) and Senior Adult Oncology Program are made up of items that have been borrowed from different geriatric domains [13,14].

The updated SIOG guidelines on the screening geriatric tests suggest that the G8 has the highest sensitivity (ranging from 65% to 92% in the different studies) [15]. It consists of eight items: one item refers to the age of the patient (<80; 80–85; >85 years) and seven items, sampled from the Mini Nutritional Assessment (MNA) questionnaire, concern nutritional status, weight loss, body mass index, motor skills, psychological status, number of medications and self-perception of health. G8 has been shown to be able to predict the chemotherapy-related toxicity and to have a prognostic role in various cancer types [16,17].

Another questionnaire, the Vulnerable Elders-13 Survey (VES-13), validated for cancer patients includes 13 items regarding the perception of health status. The test reflects the everyday activity and difficulty in performing activities related to patient health or physical condition [18]. A score ≥3 identifies patients whose health is susceptible to deterioration, with an increased risk of functional decline or death over 2 years [9].

Two studies suggest that using a combination of the two aforementioned screening tools is significantly better than the use of G8 or VES-13 alone. Therefore, the combination might be a sensitive screening tool in older cancer patients [19,20]; if these tests suggest potential “frailty”, the optimal approach to choose the tailored treatment for the patients is to perform the CGA.

In conclusion, expanding the usual clinical evaluation with quick and simple screening tests and, eventually, CGA in patients recognized as at risk could be useful to identify areas of vulnerability, predict survival and toxicities and guide clinicians in treatment decisions.

## 3. HCC Treatment in Elderly Patients According to BCLC Stage

The BCLC stage system can be used as both a prognostic and a therapeutic tool. According to the characteristics of the tumour and the degree of liver failure, patients are grouped into the following categories: very early (BCLC 0), early (BCLC A), intermediate (BCLC B), advanced (BCLC C) and terminal (BCLC D). While the BCLC stage system has mainly been developed to be incorporated into the therapeutic algorithm of HCC, BCLC categories may also provide prognostic information. However, the majority of recent prognostic studies comparing BCLC to other systems, such as MESH, ITA.LI.CA, CLIP and HKLC have clearly shown the poor prognostic performance of BCLC [21,22], underlying the disadvantages of using that stage system as prognostic. For an example, both ITA.LI.CA and CLIP score performed better than BCLC in predicting overall survival (OS) in HCC patients who were candidates for surgery [21]. Additionally, best clinical practice has clearly unveiled the limitations of BCLC treatment categories, which do not include some clinically relevant parameters, such as AFP levels, a well-known predictor of OS in various clinical situations. In this regard, a prospective study has suggested that AFP may improve the prognostic efficacy of the BCLC system, suggesting the additive effect of this biomarker [23].

An alternative approach that is useful to overcome BCLC’s drawbacks is the so-called “stage migration strategy”, according to which the treatments recommended for a different stage are selected as the best first-line treatment option [24]. Within such a treatment strategy, therapy for the next more or less advanced tumour stage may be selected, which is referred to as left-to-right or right-to-left stage migration, respectively. Stage migration is rather frequent in clinical practice: in a recent retrospective case series of 1369 HCC patients, stage migration to therapies indicated for more advanced stages was reported in approximately 60.2% of therapy choices, which is higher than previous reports [25].

“Treatment stage alternative”, which has recently been suggested by the latest American Association for the stufy of liver diseases (AASLD) guidelines [26,27], also represents a different treatment approach that is based on several therapeutic options for each BCLC stage, classified according to their level of evidence. Conversely, a “treatment hierarchy” approach is followed by both the Asia-Pacific [28] and the multi-society Italian [29] treatment algorithms. However, this strategy is completely independent of HCC staging and rather relies on other factors such as, for instance, the technical feasibility of liver resection, resulting in hierarchically organized treatments based on their effectiveness [27].

### 3.1. Early Stage HCC (BCLC Stage 0 or A)

According to BCLC classification, in patients with good health status (ECOG PS 0) and well-preserved liver function (Child-Pugh A class), solitary HCC <2 cm without vascular invasion or satellites is classified as very early stage (BCLC stage 0). In the same patients, a single tumour >2 cm or three nodules <3 cm are classified as early HCC (BCLC stage A) [30]. There is a wide range of treatment options for BCLC stages 0 or A HCC, including liver resection, liver transplantation and local ablation [30]. The BCLC classification does not specify whether age should be considered in treatment allocation, and the scientific evidence available is controversial.

#### 3.1.1. Liver Transplantation

Even though liver transplantation (LT) is superior in terms of long-term survival compared with hepatic resection or local ablation for its potential to cure both the tumour and the underlying liver disease [31], elderly patients are rarely transplanted because of low priority in a context of limited organ availability. Recent studies reported comparable survival outcomes between older and younger recipients, while others reported significantly worse survival outcomes in the elderly group [32,33,34]. The studies had a retrospective design with questionable validity, and the cut-off used for old age also varied, making direct comparison difficult. Nevertheless, the eligibility for LT should not be based only on age, but other factors must be considered in patients selection, such as the evaluation of liver function (through the model for end-stage liver disease—MELD), functional reserve (with the indocyanine green kinetics (ICG) test) and the assessment of liver stiffness (with transient elastography—LSM) [35,36,37,38]. In addition, recent studies showed that elderly HCC patients have different clinical and pathological characteristics compared to younger patients. They are more likely to have NASH-cirrhosis and HCV infections than younger patients [6,39]. They also tend to have a lower grade of background liver fibrosis and lower Child–Pugh scores compared to younger HCC patients [40]. Furthermore, they have fewer but larger HCC nodules, associated with less aggressive disease than younger patients [41,42]. These findings have to be considered and discussed within a multidisciplinary team in order to guide the patient allocation to the best-tailored treatment. However, LT as a treatment option for the aged population is still subject to debate, due to the lack of liver donors, and the upper age limit for undergoing LT is yet to be defined [43].

#### 3.1.2. Liver Resection

The older population is also under-represented in the cohort of patients undergoing liver resection for HCC [44,45], and they are usually more likely to receive conservative treatment [46]. In the majority of the studies conducted over the last twenty years, 0–14% of patients undergoing liver resection belong to the elderly population, whereas 12–28% were younger patients [44,45,46]. This fact could be likely related to the concerns regarding the increased operative risk for the older patients, with shorter expected survival [47,48].

Apart from the influence of life expectancy, elderly patients are generally characterized by higher incidences of comorbidities, such as diabetes, hypertension, cerebrovascular and cardiovascular disease, according to the studies comparing surgical resection in the elderly versus young HCC patients [49]. However, data regarding the efficacy and safety of surgical and locoregional treatments in elderly patients with HCC are limited, and current guidelines do not suggest separating treatment for older age groups [30]. The morbidity and mortality rates after hepatectomy in elderly HCC patients range from 9% to 51% and from 0% to 42.9%, respectively [46]. Due to recent advances in surgical procedures and perioperative management, most recent studies and meta-analyses have shown comparable short-term outcomes, such as hospital stay, post-operative morbidity and mortality, between younger and elderly patients undergoing hepatectomy for HCC [6,35,46,50,51,52,53]. This could probably be explained by the more careful selection criteria of elderly patients. Publication bias and heterogeneity of study design could also have led to these results.

In clinical practice, careful patient selection, irrespective of age, is crucial in achieving acceptable mortality and morbidity. Tailoring of care is appropriate, and the multidisciplinary assessment has been shown to increase the proportion of patients offered therapy with curative intent [50,51,52,53,54]. Tools that allow better selection of candidates for hepatectomy play a central role. There are different scoring systems for assessing elderly patients, including the American Society of Anesthesiologists (ASA), POSSUM/P-POSSUM, E-PASS and APACHE II score [35]. In addition, in a recent study, the use of cardiopulmonary exercise testing (CPET), enhanced recovery (ERAS) and parenchymal sparing liver surgery in the selection, management and treatment of octogenarian patients with liver tumours allowed to achieve similar post-operative outcomes to younger patients [44]. In fact, studies and meta-analyses of randomized controlled trials on the efficacy of the ERAS in liver surgery showed an improved rate of post-operative morbidity (*p* < 0.01) and length of stay (*p* < 0.01) and accelerated functional recovery (time to first flatus *p* < 0.01) [55].

The positive impact of parenchymal sparing surgery is probably related to the high incidence of hospital death due to hepatic failure after major hepatectomy in elderly patients with HCC [46]. Although the degree of liver regeneration at one month after right lobectomy is similar in younger and elderly patients, it is possible that remnant liver regeneration immediately after major hepatectomy in elderly patients is impaired [46].

In order to reduce surgical stress and improve outcomes, in the last ten years, laparoscopic and robotic techniques have been applied in liver surgery, which have been shown to be safe and effective approaches, with morbidity and mortality in major laparoscopic-robotic series in very early and early HCC of 10–15% and 1%, respectively [35,56,57,58]. Laparoscopic-robotic resection of HCC, in particular when the tumours are mainly located in superficial peripheral positions of the liver, provides optimal survival outcomes and minimizes complications and hospital stay [57,59]. Additionally, several studies have demonstrated that laparoscopic and robotic resections of HCC also in cirrhotic patients are associated with a reduced risk of post-operative liver decompensation, post-operative ascites and morbidities [57,60]. In a recent retrospective study, Wang et al. compared minor laparoscopic hepatectomy in elderly (48 patients, ≥70 years) and younger patients (97 patients, <70 years). The authors showed no significant difference in operation time, intraoperative blood loss, length of hospital stay, incidence of complications, recurrence rates, overall survival (OS) and disease-free survival (DFS) rates between the groups. Multivariate analysis showed that age was not an independent predictor of OS and DFS [61]. In another study from Korea, a propensity score matching analysis between patients ≥70 years old and <70 years old (41 patients in each arm) showed shorter hospital stay (7 versus 11 days, *p* = 0.002) in the elderly, as well as similar resections with microscopic margins involvement (so-called R1 resections) and complications rate, and no difference in 5 year OS (86.7% versus 62.2%, *p* = 0.221) and DFS (43.4% versus 30.8%, *p* = 0.500) [62]. Nomi et al. also showed a lower rate of major complications and shorter hospital stay in octogenarians with HCC treated with laparoscopic liver resection compared to younger patients [63]. For this potentially feasible and safe alternative to open hepatectomy, selecting criteria for elderly patients needs to be determined in order to benefit as many patients as possible.

Regarding long-term outcomes of liver resection, most studies showed that 5-year OS rates of elderly HCC patients after hepatectomy ranged between 26% and 75.9%, whereas those in younger HCC patients ranged from 31.4 to 68% [35,46,53]. Recent reports demonstrated comparable results between young and elderly patients for 1, 3 and 5 year OS and DFS rates [6,35,46,53,64,65,66,67,68,69,70,71,72,73,74]. In the study of Hirokawa et al., the DFS was worse in elderly patients than in the younger ones [66]. Additionally, some trials found a high prevalence of HCV infection, and a subgroup analysis showed that responders to interferon had significantly increased survival. It is well known that elderly patients respond poorly to interferon therapy due to severe side effects; nevertheless, the newer oral antiviral agents could possibly lead to improvement in DFS in elderly HCC patients with HCV infection [67].

Finally, post-operative recurrence of HCC is one of the most important factors affecting survival [68,69]. Repeated hepatectomy has also been suggested to be the most effective treatment for recurrence within the liver for elderly patients [70].

#### 3.1.3. Local Ablative Approaches

In the past decade, local ablative therapy has evolved as an alternative to resection for early-stage HCC, in particular in the case of single nodules ≤2 cm deeply or centrally located. Radiofrequency ablation (RFA) can induce coagulative necrosis of the tumour with an adequate margin (heating of tissue to 60–100 °C) [6,54]. Percutaneous RFA has been widely used and almost completely replaced other local treatments, such as PEI (percutaneous ethanol injection) and PAI (percutaneous acetic acid injection). In a network meta-analysis of treatment of early HCC, including 2096 patients in 14 randomized clinical trials, RFA caused larger ablation volume in fewer sessions of treatment and better OS and DFS compared to PEI and PAI [71]. For very early HCC, the European Association for the Study of the Liver (EASL) guidelines suggest considering RFA as a first-line option, leaving surgery to those patients who fail treatment or with nodules that are not suitable for RFA [30]. In a recent meta-analysis, subgroup analysis according to patient’s age (65–75 vs. >75 years) and tumour size (≤3 vs. 3–5 cm) revealed that liver resection achieved better OS than RFA, except for patients older than 75 years with tumours ≤3 cm [72]. In a Japanese trial, Kaibori et al. used a propensity score analysis to match elderly patients treated with RFA or liver resection with similar liver function and tumour characteristics [73]. They showed that liver resection decreased recurrence risk and improved OS in patients >75 years with HCC <3 cm. Another meta-analysis comparing surgical outcomes, quality of life and costs between patients with HCC treated with RFA or liver resection concluded that, for very early HCC (single nodule <2 cm) in Child–Pugh class A patients, RFA provided similar life expectancy and quality-adjusted life expectancy at a lower cost [74]. Another retrospective study by Jiang et al. concluded that in elderly patients, RFA should be recommended for those with HCCs ≤ 2 cm, while surgical resection would be a better treatment for those with HCCs of 2–5 cm [75].

Significant advantages of surgical resection are mainly attributable to the removal of potential venous tumour thrombi, complete eradication of the primary tumour with clean resection margins and the possibility to have information about histopathology [30]. The presence of satellites, microvascular invasion or poor differentiation could guide the clinician in the post-operative management, including the possibility to consider an “ab initio” liver transplantation [76]. Given that elderly patients are generally not candidates for liver transplantation, the availability of the pathology characteristics does not usually affect the treatment strategy for this group of patients.

In addition, elderly patients have more comorbidities and may be poorer candidates for liver surgery. For these reasons, RFA has many advantages, such as minimal invasiveness, less blood loss, lower perioperative risk, and fewer negative effects on liver function. Therefore, RFA may be more feasible than surgical resection in elderly patients [72].

Recent studies and meta-analyses did not demonstrate any significant differences in terms of duration of hospitalization and serious adverse events among the elderly HCC patients and their younger counterparts treated with RFA [6,77]. The most common complications were hemoperitoneum, liver abscess, hemothorax, subcutaneous hematoma, and asymptomatic biloma. Less common complications included pneumothorax, hemobilia, massive hepatic infarction and gastrointestinal perforation [6].

Regarding the outcomes, several studies have reported five-year survival beyond 70% in well-selected patients with very early HCC treated with RFA [30]. In a recent meta-analysis, Hung et al. showed no significant differences in 1-year (odd ratio (OR) = 1.5, 95% confidence interval (CI): 0.788–2.885, *p* = 0.217) and 3-year OS (OR = 1.352, 95% CI: 0.940–1.944, *p* = 0.104) in elderly and younger patients who underwent RFA for HCC. At 5 years, the younger patients had significantly better clinical outcomes (OR = 1.379, 95% CI: 1.079–1.763, *p* = 0.01) [6]. Fujiwara et al. showed higher mortality at 5 years in elderly patients. However, an additional risk analysis demonstrated that there was a significant difference in liver-unrelated deaths between the elderly and younger patients [78]. This suggests that elderly patients tend to die from liver-unrelated causes at 5 years. Nishikawa et al. demonstrated decreased cumulative OS and DFS at 1-, 3- and 5-year intervals in the elderly patient group [79].

Nevertheless, RFA has a significant drawback of limited ablative margins, which is associated with a high risk of marginal recurrence [31]. This is particularly true in HCC > 3 cm as shown in previous studies, in which tumour size has been identified as an independent risk factor for local recurrence and OS [31,80,81,82]. In addition, RFA may not eradicate the tumour effectively in subcapsular HCC, or close to major bile ducts, large vessels or intestine. This may be associated with a higher risk of complications and recurrence, resulting in worse survival [83,84].

Although percutaneous RFA is the least invasive procedure, for tumours in difficult anatomical locations many centers have adopted laparoscopic radiofrequency ablation (LRFA). Two of the advantages of using this technique, compared with percutaneous RFA, are the real-time security monitoring of the ablation process and the accurate detection of tiny lesions [30]. However, a meta-analysis comparing LRFA to liver resection for early HCC (1691 patients in 11 studies) showed that patients undergoing hepatic resection had higher 3- and 5-year OS rates, higher 3-year DFS rate, and lower local recurrence rate than those undergoing LRFA. Nevertheless, patients undergoing LRFA had higher 3- and 5-year OS rates than those undergoing other minimally invasive ablation, although there was no statistical difference in local recurrence or DFS rate [85].

Interest in microwave ablation (MWA) has increased in recent years due to its potential physical advantages, which have been facilitated by modern high-powered devices [73]. In fact, MWA uses high-frequency microwave energy to heat the tumour to 60–100 °C and causes cell coagulation necrosis [86]. It is considered more effective than RFA in inducing higher intra-tumoural temperature, greater tumour ablation volume and faster ablation times, and it has a better convection profile than RFA [87]. However, complications such as liver failure, bleeding, infection, abscess, intercostal nerve injury, bile duct stenosis, organ injury and pneumothorax can develop at a rate of 2–3% [87,88]. It was reported that MWA could be as effective as RFA for single HCC < 3 cm and could have better tumour inactivation ability over RFA for 3–5 cm tumours and tumours adjacent to vessels and gallbladder [88,89].

A large study from China reported outcomes after MWA in 1007 patients with HCC. The 1- and 5-year survival rates were 91.2% and 59.8%, respectively. Subgroup analysis of the study indicated a 5-year survival rate of 29–68.6% for those with lesions >5 cm [90,91]. A recent meta-analysis comparing RFA and MWA therapy showed similar rates of complete response and local recurrence, with lower local recurrence rates in those with larger nodules treated with MWA and a lower 3-year survival rate, without statistical significance, compared to RFA. Major complications occurred more frequently with MWA than with RFA [92]. In a systematic review and meta-analysis including 26 studies (5 randomized clinical trials and 21 cohorts), the outcomes of 2393 patients treated with RFA were compared to 2003 patients treated with MWA [86]. Forty-seven percent of patients received treatment under general anesthesia in the MWA group and 84% in the RFA group (OR = 0.529, *p* < 0.001). The median ablation time was reduced in the MWA group (12 min) compared with the RFA group (29 min) (*p* < 0.001). In total, 17.6% patients exhibited progression during follow-up in the MWA group compared with 19.5% in the RFA group (OR = 0.877, *p* = 0.225). No statistically significant differences were observed between MWA and RFA groups in terms of OS and DFS (hazard ratio (HR) = 0.891 and 1.014, *p* = 0.222 and 0.852, respectively). One study that reported clinical outcomes after MWA suggested that this treatment option is safe and effective for older patients with HCC (>65 years) [93]. Finally, in a recent study, 510 elderly (≥65 years) and 1053 younger patients (<65 years) were diagnosed with early-stage HCC according to the Milan criteria. Similar survival outcomes were obtained in elderly and younger HCC patients treated by MWA, despite elderly patients having more comorbidities [94].

Data on other techniques, such as laser ablation, cryoablation and irreversible electroporation (IRE) are limited, and further studies are required in this field [95,96,97].

### 3.2. Intermediate-Stage HCC (BCLC Stage B)

According to current clinical guidelines, transcatheter arterial chemoembolization (TACE) is the recommended treatment for intermediate-stage HCC (multifocal, unresectable HCC, without macrovascular invasion or extrahepatic metastasis) patients with preserved liver function and absence of physical impairment (ECOG PS 0) [4,98]. These patients, classified as BCLC stage B, represent a heterogeneous population with different tumour burdens and, consequently, different survival rates after TACE. The median OS expected in the best responders is around 36–45 months versus 11 months in the patients who do not show a response to treatment. The American and European guidelines do not provide precise recommendations about the number of TACE cycles to use in this setting before switching to systemic therapy [4,98,99,100].

In this context, the subclassification of the intermediate stage defining specific treatment for each subgroup and the identification of prognostic scores of patients’ stratification after TACE are topics of great interest, and much work is being done about them. In 2012, Bolondi et al. [100] proposed a sub-classification of stage B, which included four stages (B1, B2, B3, B4) and, in addition, another stage defined “quasi C”. These stages were identified according to four parameters: Child–Pugh score, “Beyond Milan” and “within up to seven” criteria (defined as HCC with 7 as the sum of the diameter of the largest tumour (in cm) and the number of tumours), ECOG performance status and presence (or absence) of portal vein tumour thrombosis. For each stage, the authors matched a first treatment option, an alternative option and a median survival time.

For the B1 substage, TACE was indicated as the first option, reserving the transplantation for patients who met the up-to-seven criteria; for B2 patients, TACE or Y-radioembolization (TARE) in the first instance and sorafenib as an alternative option; for B3 patients, clinical trial or TACE/sorafenib; for B4 patients, liver transplantation if they met the up-to-seven-criteria and sorafenib for stage “Quasi C”.

The median survivals of these subgroups were different: 41.0 months in stage B1, 22.1 months in stage B2 and 14.1 months in stage B3, confirming the assumed prognostic value of sub-classification in relation to treatment choice. Later, Kudo et al. proposed the “kinki criteria”, distinguishing the patients in the already mentioned three stages (B1-B2-B3) [101,102] but considering only the Child–Pugh score (5–7 or 8–9) and the “Beyond Milan” + “within up to seven” criteria (yes or no), in a more useful way for clinical practice.

Although these systems need external validation in terms of prognostic ability, they highlighted that patients with substage B2 HCC, with bilobar multiple HCC, beyond the up-to-seven criteria, are those that could be considered for a first line with sorafenib, as they could easily become TACE-refractory.

The other attractive aspect of the intermediate stage is the re-treatment algorithm in patients with TACE failure. In this regard, the Assessment for Retreatment with TACE (ART) score was externally validated [103], and higher ART score values (≥2.5 prior to the second TACE) were associated with major adverse events after the second TACE (*p* = 0.011), identifying patients who may not benefit from further TACE. However, to date, only hepatoma arterial-embolization prognostic (HAP) score [104] has been validated in a prospective trial; it performed better than other scoring systems in differentiating high- and low-risk groups. The HAP score is another predictor of outcomes of patients with HCC undergoing TACE/TAE.

In the elderly population, TACE confirms the clinical outcome and safety profile already shown in the younger (<70 years old). In 1994, Mondazzi et al. showed that disease stage (tumour stage, alphafetoprotein value, hepatic functional reserve) was the main independent prognostic factor of survival in elderly patients, whereas there was no correlation with the chronological age [105]. Then, Yau et al., in a large Korean study, showed an increase in survival in patients older than 70 years compared to younger patients (median OS 14 months versus 8 months), even if the two populations were unbalanced by baseline characteristics [106]. Additionally, TACE was well tolerated in both groups (elderly and non-elderly patients) in retrospective studies and meta-analysis, including observational cohorts [6]. Although there is a lack of randomized controlled trials, based on the available data in the literature, the cumulative risk of both liver-related death and PFS after TACE is comparable in “elderly” and “younger” patients. Therefore, TACE remains a preferred choice in elderly patients even when they meet criteria for resection, due to the safety of TACE and the higher risk of surgical complications in this population [107,108]. In this regard, Liu et al. empathized this aspect, reporting that 67% of old patients underwent surgical resection, compared with 74% of younger patients [109].

Regarding the use of subsequent TACE in elderly patients after disease recurrence, international guidelines do not recommend the procedure due to the high risk of AEs, especially gastrointestinal ones (nausea, vomiting, diarrhea) [4,98]. Therefore, the use of systemic therapy might be considered after the first TACE failure with a median OS ranging between 11 and 14 months in this population [110,111].

In conclusion, the choice of the optimal treatment strategy in elderly patients with intermediate HCC stage depends on the cancer stage and—most importantly—on the assessment of general clinical conditions in a multidisciplinary context. Based on the data available in the literature, there is no difference in the optimal treatment according to age. However, larger and further prospective studies are needed to confirm these results in this heterogeneous population.

### 3.3. Advanced Stage HCC (BCLC Stage C)

According to current international clinical guidelines, advanced-stage HCC patients, which include subjects showing vascular involvement/extrahepatic spread (N1 and/or M1) or physical impairment (ECOG PS 1–2), are candidates for systemic therapy [4,98].

Table 1 summarizes the most important data in the literature regarding systemic treatment in elderly patients with advanced HCC, in both the first- and second-line settings.

#### 3.3.1. First-Line Treatment

To date, FDA has approved sorafenib, lenvatinib and atezolizumab plus bevacizumab as a first-line therapy, whilst European Medical Agency (EMA) has approved only the first two drugs [120,121].

Sorafenib was the first drug approved for advanced HCC patients. It is an oral multikinase inhibitor of serine/threonine-kinases *CRAF* and *BRAF*, but also of transmembrane receptors such as vascular endothelial growth factor receptor 2 (*VEGFR2*) and 3 (*VEGFR3*), platelet-derived growth factor receptor (*PDGFR*), *c-KIT* (or *CD117*), *FLT3* (or *CD135*) and *RET* [122]. Concerning pharmacokinetics, sorafenib tablets are administered 400 mg bis in die, with a discrete bioavailability (<50%); it is metabolized by hepatic enzymes such as *CYP3A4* and *UGT1A9*, and then its glucuronide conjugate is mainly excreted via the biliary/fecal route [123]. The main AEs are diarrhea, fatigue, hand–foot skin reaction (HFSR) and hypertension [124]. Sorafenib was approved by the FDA in 2007 on the basis of the results of two phase III clinical trials: SHARP, conducted in western countries, and the Sorafenib Asia-Pacific both demonstrated a meaningful prolongation in OS by using sorafenib versus placebo [112,113]. In a combined analysis of the two trials, there was no difference in OS between patients younger or older than 75 years (*p* = 0.6304) [125].

A recent international cohort study on 5598 HCC patients treated with sorafenib, including 792 patients aged ≥75 years, tried to answer several issues concerning elderly patients [126]. The trial confirmed similar efficacy of treatment in both patient groups as well as a similar rate in grade 2–4 AEs (63.5 versus 56.7%, *p* = 0.11); however, elderly patients showed a slightly higher rate of discontinuation due to AEs (27.0 versus 21.6%, *p* < 0.01). Additionally, in this retrospective analysis, a lower starting dose of sorafenib (200 or 400 mg die), which is a consolidated but not formally recommended clinical practice, did not affect survival in patients over 75 years [127]. Therefore, this dosage could be considered as an option in those patients, especially if they have had some AEs, in order to proceed with the therapy.

Lenvatinib is an oral multikinase inhibitor of *VEGFR 1–3*, fibroblast growth factor receptors 1-4 (*FGFR1-4*), *PDGFR*α, *RET* and *c-KIT*, thus showing a similar pharmacodynamics profile to sorafenib [128]. Lenvatinib’s daily dose differs according to bodyweight—8 mg if <60 kg, 16 mg if ≥60 kg; it is metabolized by *CYP3A4* and excreted in both stool and urine [129]. Lenvatinib was approved by FDA in 2018, relying on results of the non-inferiority phase III REFLECT trial, which shows similar efficacy data compared with the standard of care sorafenib, but there was a different safety profile between the two drugs: less frequent diarrhea and HFSR for lenvatinib, but more frequent hypertension, proteinuria and fatigue than the control arm [111]. In this trial, ~30% of patients were aged 65–75 years and ~13% were aged ≥75 years; no difference between patients aged <65 and ≥65 years was reported in terms of both progression-free survival (PFS) (HR: 0.67 versus 0.61) and OS (HR: 0.94 versus 0.84), according to non-preplanned subgroup analysis. Then, a propensity score matching analysis from a cohort of patients who received lenvatinib as a first-line treatment showed that patients aged ≥75 years had similar efficacy but different rates of AEs compared to those aged <70 years, with HFSR being less frequent in the elderly (22% versus 42%, *p* = 0.053). However, HFSR occurrence was associated with an increased OS [130].

The recently approved association of atezolizumab—a monoclonal antibody directed against the programmed death ligand 1 (PD-L1)—and bevacizumab—a monoclonal antibody directed against *VEGF*—has been tested versus sorafenib in a phase III trial as a first-line treatment for advanced HCC, showing a statistically significant improvement in OS (HR: 0.59; *p* < 0.001) [110]. An exploratory subgroup analysis from this study pointed out a similar survival and AEs rate in patients younger or older than 65 years, with a slightly higher incidence of AEs leading to dose interruption [131].

In conclusion, to date, all these therapeutic options appear to be effective and safe in elderly people affected by advanced HCC, although a careful geriatric assessment should be performed before starting treatment in order to identify points of potential weakness, i.e., expected impact of specific adverse events on everyday life activities.

#### 3.3.2. Second-Line Treatment

To date, the FDA has approved regorafenib, cabozantinib, ramucirumab, nivolumab and pembrolizumab as second-line therapy for advanced HCC patients, whilst EMA has not yet approved the last two drugs [132,133,134,135,136,137,138,139].

Regorafenib is an oral multikinase inhibitor of *VEGFR 1-3, TIE-2, PDGFR, FGFR, c-KIT, RET* and *BRAF* [140] administered at a daily dose of 160 mg; it is converted by *CYP3A4* and *UGT1A9* in its pharmacologically active metabolites M-2 and M-5, which undergoes enterohepatic circulation [141,142]. The phase III RESORCE trial randomized HCC patients who progressed after sorafenib to regorafenib once daily for 21 consecutive days of each 28-day cycle or placebo. Patients receiving regorafenb had a meaningful benefit in OS (HR: 0.63, *p* < 0.0001), and the efficacy of regorafenib in patients ≥ 65 years was comparable to that observed in the overall population, with an HR of 0.74 in the subgroup analysis [116]. In this trial, the main AEs were HFSR, diarrhea, fatigue, hypertension and increased blood bilirubin. No further data about elderly HCC patients treated with regorafenib have been reported to date.

Cabozantinib is another multikinase inhibitor, targeting *VEGFR2*, *RET*, *KIT*, *AXL*, *TIE2*, *ROS1*, *TRKB*, and *FLT3*, which shows a pharmacokinetic profile similar to lenvatinib [143]. In a phase III CELESTIAL trial, cabozantinib was compared with placebo in HCC patients who progressed after at least one systemic treatment; half of the enrolled population was ≥65 years old [117]. Diarrhea, decreased appetite, HFSR, fatigue, nausea and hypertension are major AEs. A sub-analysis of this trial in patients >65 years showed that patients receiving cabozantinib had a benefit in both OS and PFS compared to younger ones (HR: 0.74 versus 0.81 and HR: 0.46 versus 0.45, respectively). Adverse events, good quality of life—based on similar rate of dose reductions—and patients receiving subsequent therapies were similar in elderly and younger patients [144].

Ramucirumab is a monoclonal antibody-blocking *VEGFR2* activity, which demonstrated activity in HCC patients who progressed to sorafenib, when α-fetoprotein concentrations were ≥ 400 ng/mL, according to REACH-2 trial results [119]. Among AEs, peculiar ones are bleeding or hemorrhage events, proteinuria, hypertension and epistaxis. A recent pooled analysis of REACH and REACH-2 trials showed no difference in both efficacy and safety between patients <65 years, 65–75 years and >75 years treated with ramucirumab, with a remarkable median relative dose intensity greater than 97% [145].

Nivolumab and pembrolizumab are both anti-PD-1 monoclonal antibodies; they inhibit the block due to PD-1/PD-L1 cooperation, resulting in an increased T-cell cytotoxic activity. They have been tested as second-line treatment in HCC patients in two phase II clinical trials, showing a manageable safety profile [146,147]. However, specific data about elderly patients treated with immune-checkpoint inhibitors have not been reported yet.

#### 3.3.3. Comorbidities and Contraindications

Treatment of advanced HCC in elderly patients could be challenging because of comorbidities, which affect anticancer drug delivery. It must be noted that the above-mentioned clinical trials did not include patients with comorbidities that are indeed frequent in the elderly as well as patients with poor ECOG PS (ECOG PS 2). In fact, a history of cardiac disease was an exclusion criterion in SHARP and REFLECT trials, as well as a history of bleeding or thrombotic disorders or use of anticoagulants, which are major contraindications for anti-angiogenetic multikinase inhibitors [110,114]. Renal impairment is not a contraindication for sorafenib administration except if it is severe (requiring hemo- or peritoneal dialysis). For oral drugs, malabsorption should also be considered [148].

Special attention should be paid to the concomitant medications, given the high frequency of comorbidities requiring drug intake in the elderly. Concomitant medications could affect oral multikinase inhibitor efficacy at several levels, i.e., by reducing their absorption or by favoring their elimination; however, major concerns refer to metabolic interaction via CYP enzymes: CYP inducers or inhibitors could hamper metabolic activation or inactivation of several anticancer drugs. Additionally, CYP expression seems to be decreased in HCC tissue; this fact, associated with a physiologic reduction due to ageing, could severely reverberate on the efficacy of multikinase inhibitors in the elderly or on AEs onset in HCC patients [149,150,151].

Concerning hepatic impairment in treatment-naïve HCC patients who are candidates for multikinase inhibitors, Child–Pugh B was an exclusion criterion in SHARP, Asia-Pacific and REFLECT trials, but some patients were included as a result of protocol violation (<3%) [110,111,112]. In the second and further lines of treatment, multikinase inhibitors have been tested only in Child–Pugh A patients, meaning that HCC patients who switch from Child–Pugh A to B due to progressive disease have a loss of therapeutic chances. Immunotherapy trials in the second-line setting excluded Child–Pugh B patients. However, also in these trials, some patients were improperly included; a recent Korean real-world cohort study of nivolumab in HCC patients pointed out that Child–Pugh B is an independent negative predictor for objective response, with worse OS if compared to Child–Pugh A [152].

Child–Pugh C is an assured exclusion criterion for all drugs: patients belonging to this class must receive best supportive care only. A retrospective analysis of 992 Korean HCC patients showed no difference in percentage of Child–Pugh classes between patients older and younger than 70 years, and a similar result has been reported in extremely elderly patients (>80 years) in a Japanese cohort [153,154].

Even if data specifically on elderly HCC patients are lacking, immunotherapy seems to be feasible in this population without major contraindications (except for active autoimmune diseases), paying attention to steroid administration, which could reduce its efficacy.

#### 3.3.4. A Focus on Sarcopenia in Elderly HCC Patients

One of the most important assessments in elderly patients is the nutritional one due to the higher rate of sarcopenia in this population. In particular, sarcopenia consists of a progressive skeletal muscle wasting and weakness due to due to loss of muscle quantity and quality, typically associated with age and often being part of cancer cachexia syndrome [155]. Concerning HCC patients, a Japanese retrospective analysis showed a 35% prevalence of sarcopenia in elderly patients (>70 years), with lower serum albumin levels and lower nutrition index, conferring a poor prognosis [156]. Sarcopenic patients undergoing sorafenib treatment had a significantly decreased OS (39 weeks compared to 61 weeks in non-sarcopenic patients) in an Italian retrospective analysis; sarcopenia might be enhanced by sorafenib administration through a reduction in carnitine absorption, suggesting that patients could benefit from carnitine integration [157,158]. Carnitine deficiency has been also observed in patients who developed fatigue during lenvatinib treatment [159].

Overall, these data highlight the role of muscle metabolism in HCC patients and especially in older ones, showing that improving the muscular status of these patients could be advantageous in their clinical management, particularly in advanced stages where supportive therapy plays a fundamental role [160].

## 4. Conclusions

HCC is a complex illness to manage, especially in the elderly, and even today there is a lack of prospective evidence in the literature or treatment guidelines in this population. The increase in life expectance and the peak of HCC incidence in older age enhanced the number of elderly HCC patients. Therefore, the management of elderly patients with HCC is a largely unexplored field worth investigating in clinical trials. In general, these patients should be referred to dedicated, high-volume centers with specialized tasks.

HCC patients require a comprehensive multidisciplinary—including geriatric—assessment, in order to receive correct tumour staging and a careful evaluation of the abilities and comorbidities. Finally, elderly patients should be distinguished into “frail” and “fit” patients. Even if specific data do not exist in the literature to date, the evidence summarized in this review suggests the use of geriatric scales in everyday clinical practice. In particular, fit patients could receive the best standard treatment for HCC according to BCLC stage, regardless the chronological age [4,98], which should not represent one of the criteria to select patients for treatments. Additionally, also in the geriatric population, the novel concept of stage migration is becoming an important issue to consider in order to give them the opportunity to receive a treatment different from that indicated by BCLC because of its clinical characteristics. Thus, an in-depth evaluation of the risk/benefit of different treatments should be especially mandatory in this population.

**Figure 2:**
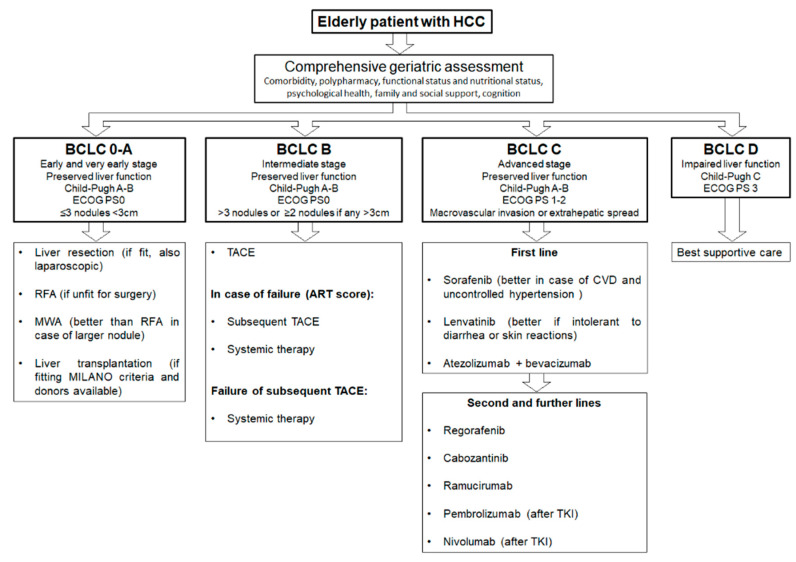
Proposed algorithm for treating elderly hepatocellular carcinoma (HCC) patients according to Barcelona Clinic Liver Cancer (BCLC) stages.

Finally, in the case of frail elderly patients, they should be candidates for the best supportive care and eventually referred to the palliative care unit. However, more specific and prospective data are needed in this field, and further investigations are required in order to standardize the treatment for HCC elderly patients. In Figure 2, we propose a summary algorithm for treating HCC in elderly patients.

## Figures and Tables

**Table 1:** Landmark trials for advanced HCC treatment in the first- and second-line settings with a focus on the elderly patients.

Trial	Drug(s)	Primary Endpoint	Elderly (%)	Elderly (Efficacy)
**First line**				
**SHARP [112]**	Sorafenib vs. placebo	OS: 10.7 vs. 7.9 months (*p* < 0.001)	Combined analysis: ≥75 y: 16.8%	Combined analysis: <75 y vs. ≥75 y: no difference (*p* = 0.6304)
**Asia-Pacific [113]**	Sorafenib vs. placebo	OS: 6.5 vs. 4.2 months (*p* = 0.014)		
**REFLECT [114]**	Lenvatinib vs. sorafenib	OS: 13.6 vs. 12.3 months (non-inferior)	65–75 y: 30%≥75 y: 13%	PFS <65 vs. ≥65 y: no difference OS <65 vs. ≥65 y: no difference
**IMbrave150 [115]**	Atezolizumab + bevacizumab vs. sorafenib	1y-OS: 67.2% vs. 54.6% (*p* < 0.001)	NR	OS <65 vs. ≥65 y: no difference
**Second line**				
**RESORCE [116]**	Regorafenib vs. placebo	OS: 10.6 vs. 7.8 months (*p* < 0.0001)	≥65 y: 45%	OS ≥65 y: comparable to overall population
**CELESTIAL [117]**	Cabozantinib vs. placebo	OS: 10.2 vs. 8 months (*p* = 0.005)	≥65 y: 48.5%	PFS <65 vs. ≥65 y: no difference OS <65 vs. ≥65 y: no difference
**REACH [118]**	Ramucirumab vs. placebo	OS: 9.2 vs. 7.6 months (*p* = 0.14)	Pooled analysis: 65–75 y: 18.6% ≥75 y: 9.3%	Pooled analysis: OS <65 vs. 65–75 vs. ≥75 y: no difference
**REACH-2 [119]**	Ramucirumab vs. placebo	OS: 8.5 vs. 7.3 months (*p* = 0.019)		

Abbreviations: OS: overall survival; y: years old; PFS: progression-free survival; 1y: one year; NR: not reached.
